# The Beverage Hydration Index: Influence of Electrolytes, Carbohydrate and Protein

**DOI:** 10.3390/nu13092933

**Published:** 2021-08-25

**Authors:** Mindy Millard-Stafford, Teresa K. Snow, Michael L. Jones, HyunGyu Suh

**Affiliations:** Exercise Physiology Laboratory, Georgia Institute of Technology, Atlanta, GA 30332, USA; teresa.snow@ap.gatech.edu (T.K.S.); mjones46@gatech.edu (M.L.J.); hyungyu.suh@gatech.edu (H.S.)

**Keywords:** sodium, potassium, fluid balance, sports drink, dipeptide, osmolality

## Abstract

The beverage hydration index (BHI) facilitates a comparison of relative hydration properties of beverages using water as the standard. The additive effects of electrolytes, carbohydrate, and protein on rehydration were assessed using BHI. Nineteen healthy young adults completed four test sessions in randomized order: deionized water (W), electrolytes only (E), carbohydrate-electrolytes (C + E), and 2 g/L dipeptide (alanyl-glutamine)-electrolytes (AG + E). One liter of beverage was consumed, after which urine and body mass were obtained every 60 min through 240 min. Compared to W, BHI was higher (*p* = 0.007) for C + E (1.15 ± 0.17) after 120 min and for AG + E (*p* = 0.021) at 240 min (1.15 ± 0.20). BHI did not differ (*p* > 0.05) among E, C + E, or AG + E; however, E contributed the greatest absolute net effect (>12%) on BHI relative to W. Net fluid balance was lower for W (*p* = 0.048) compared to C + E and AG + E after 120 min. AG + E and E elicited higher (*p* < 0.001) overall urine osmolality vs. W. W also elicited greater reports of stomach bloating (*p* = 0.02) compared to AG + E and C + E. The addition of electrolytes alone (in the range of sports drinks) did not consistently improve BHI versus water; however, the combination with carbohydrate or dipeptides increased fluid retention, although this occurred earlier for the sports drink than the dipeptide beverage. Electrolyte content appears to make the largest contribution in hydration properties of beverages for young adults when consumed at rest.

## 1. Introduction

Water is considered the most essential nutrient. Yet, achieving an adequate total daily water intake may vary due to a number of factors, including the environmental temperature and the individual’s physical activity, body composition, age, and food/fluid intake [1]. Beverages contribute approximately 80% of total water intake each day and thus are considered a primary vehicle for maintaining hydration status [2]. In addition to drinking water, it has been recognized that different beverages provide fluid to the body under most conditions. For example, the addition of salt to ingested fluids directly improves fluid retention, particularly when restoring fluid balance after significant electrolyte loss occurs via sweating elicited by exercise [3,4,5]. Moreover, the inclusion of other macronutrients (e.g., carbohydrate, protein) in athletes further benefits the restoration of fluid balance [6,7,8,9] in addition to facilitating recovery from exercise by replenishing muscle glycogen and aiding protein re-synthesis, respectively [10].

However, the maintenance of an optimal hydration status is also of interest in the general population and not necessarily related to rehydration following exercise. The water content of beverages enters the body water pool at a rate dictated by several physiological processes, including gastric emptying and intestinal absorption [11], and is then subsequently lost by various routes, primarily urine (in the absence of sweating). Among other individual physiological factors, fluid retention appears related to the age of individuals; beverage composition, notably macronutrient/electrolyte content; and substances that increase diuresis (e.g., alcohol, medications such as furosemide) [12,13,14]. In order to directly compare the short-term hydration properties of common beverages, Maughan et al. [15] developed the beverage hydration index (BHI) model. BHI assesses the hydration potential of a consumable fluid relative to plain water when individuals are in a resting state and assumes that a beverage with greater diuresis relative to water has less available fluid retained in the total body water pool (and a BHI index below 1.0). Although a relatively new metric for beverages analogous to a scale comparing the glycemic index for foods [15], the BHI has since been replicated by several other groups [14,16,17,18,19]. Notably, the impact of population-specific variables, such as body mass and sex, appear minimal, and the reproducibility of BHI is reportedly robust [19]. Thus, the BHI has received attention as a valid model to assess beverage hydration characteristics under well-controlled conditions when individuals are euhydrated (in contrast with rehydration protocols following exercise).

The impact of adding electrolytes to water appears to result in a greater fluid retention when using the BHI method [13,18]. However, the minimum level of sodium to accomplish this is not consistent across studies since several report the BHI of sports drinks (~20 mmol sodium) does not necessarily yield a significantly greater BHI compared to the water control [15,16,19]. This point bears additional investigation since sports drinks are frequently touted for general public use as a viable beverage for oral rehydration following dehydration. Beverages that contain higher sodium (typically ≥45 mmol), such as Pedialyte, are observed to have higher BHI relative to water, although Pedialyte may or may not have a BHI superior to a sports drink [14,15,19]. Such beverages (e.g., Pedialyte) are often considered comparable to an Oral Rehydration Solution (ORS), although it is not entirely clear if these beverages meet the strict ORS definition of containing higher sodium (75 mmol), which improves fluid retention over water [20].

Emerging evidence suggests other components within an ORS base formula might include amino acids. The small intestine has membrane transporters depending on the amino acid or peptide ingested, some of which co-transport sodium. For example, glutamine or alanine appear to potentially increase electrolyte/water absorption following acute infection [21,22] or exercise [23]. Substituting amino acids for carbohydrate in ORS has recently been suggested as a potential advantage for ORS [14,19] due to the carrier-mediated amino acid transport for intestinal absorption of sodium and water but without the added calories from sugars in the beverage [17]. This might prove particularly advantageous in clinical populations (i.e., diabetic or obese individuals). However, these previous studies, which have examined a blend of up to eight different amino acids [14,19] using the BHI model and reporting higher fluid retention relative to water, also contained sodium concentrations over twice that of the other beverages compared within the same experiment. Therefore, the impact of specific amino acids versus the sodium per se are unclear in evaluating the optimal composition of various beverages for promoting fluid retention. Thus, the potential additive benefit of specific nutrients (electrolytes, electrolytes plus carbohydrate or protein) needs to be systematically evaluated within the same group of subjects performing the BHI.

Therefore, our purpose was to control the composition of electrolytes in order to examine the additive effects of carbohydrate (similar to a concentration typically found in sport drinks) versus a modest addition of protein (alanyl-glutamine dipeptide). We hypothesized that while adding electrolytes to plain water should theoretically improve the BHI, the supplementary addition of macronutrients (i.e., carbohydrate or dipeptide) would further contribute to raising the BHI index over 4 h following beverage ingestion. Achieving an equivalent BHI when substituting a dipeptide for sugar in a beverage without sacrificing either taste or acceptability could prove advantageous for the general population as well as those individuals requiring reduced sugar intake.

## 2. Materials and Methods

### 2.1. Study Population

Nineteen young adult, non-obese men and women from the local campus and Atlanta community completed the study. One woman was unable to complete all trials due to the human research shutdown during the global pandemic, and these data are not included in the analysis. Exclusion criteria included a baseline body composition to screen for obesity (>35% body fat for women, >30% for men). Participants were excluded who had an active infection (e.g., urinary tract); cardiovascular, metabolic (e.g., diabetes), renal, endocrine (hypo-, hyper-thyroid), or digestive diseases (e.g., hepatitis, cirrhosis, irritable bowel); smoking history and/or use of nicotine-containing products; illegal/recreational drug use; any medications that alter fluid balance (e.g., diuretics); or over-counter drugs (e.g., anti-histamines, stimulants). Participants were excluded if they used any dietary supplements during the study (except a multi-vitamin-mineral supplement—100% of recommended daily amount or less). Participants were not excluded due to caffeine intake (naïve or habitual users), but caffeine was restricted on the day of the test protocol along with alcohol-containing beverages. Women did not participate if they were pregnant or believed they might possibly be pregnant. No participants were on an active weight loss program during the study.

During a familiarization visit to the lab, participants had height, weight, and body composition assessed using the seven-site skinfold method for men and women [24,25]. Total body water was estimated using a multi-frequency bioimpedance instrument (InBody 770, Seoul, South Korea). Total body water was also estimated [26] based on fat free mass obtained with skinfolds. Body composition was used only to evaluate the exclusion criteria. The study was approved by the Institutional Review Board at Georgia Tech prior to subjects giving written informed consent. Although 21 subjects were enrolled prior to the research shutdown due to the COVID-19 pandemic, only 19 subjects completed the study. Two women were unable to complete the study six months later after human research was permitted to resume. Table 1 indicates the mean (± standard deviation, SD) physical characteristics of participants who completed all four trials

### 2.2. Study Design

All testing was performed in the Exercise Physiology Laboratory at Georgia Institute of Technology in Atlanta, GA, USA. This study was a randomized, double-blind (except for the water control trial), crossover study with each subject serving as their own control by completing all four beverage conditions. To ensure the four beverage treatments would be allocated in a counterbalanced order, a Latin Square design was determined a priori by the study coordinator with subjects assigned randomly following enrollment. Water and the 3 test drinks were nearly equally distributed (based on total subjects completing all trials) to receive an equal number of times in the 1st through 4th order position. All trials began between 0700–0900 h, and the time of day for testing was held constant for each subject. Each test session was conducted in thermoneutral conditions (21°C) and separated by a minimum of four days apart. Subjects refrained from strenuous activity for the 24 h before each test day and recorded their 24 h dietary intake (including all beverages). Participants were also asked to replicate a similar dietary intake on subsequent visits. In addition, water intake was prescribed for 16 h prior to beginning the experimental session. 

### 2.3. Test Beverages

The test beverages were: deionized water (W), sugar-free electrolyte drink (E), sports drink (5.8% carbohydrate: 2.6 g glucose/3.2 g fructose per 100 mL) with electrolytes (C + E), and alanyl-glutamine dipeptide (2 g/L) drink with electrolytes (AG + E). The rationale for using an alanyl-glutamine dipeptide was based on previous studies indicating potential efficacy in a rehydration beverage following exercise [23] and other clinical models [21,22]. All beverages except W control were received blinded (identified only by numerical codes). Beverage composition is presented in Table 2. The test beverages had similar coloring, flavoring, and electrolyte/vitamin contents. Other nutrients in the test beverages (except for W) included 5.1 mg/L Mg^+^, 12.9 mg/L Ca^+^, niacin, and vitamins B6 and B12 (equivalent to 15% daily value per 355 mL). Beverage osmolality, sodium, and potassium were directly measured after the conclusion of the experimental trials. Osmolality was measured in triplicate with 50 µL samples by freezing-point depression using a Micro-Osmette osmometer (Precision Systems, Inc, Natick, MA, USA). Electrolytes were measured with Horiba LAQUA twin (Horiba, Kyoto, Japan) compact ion meters. Beverages were stored in a refrigerator for the duration of the study and up through beverage measurements at 5 °C. All beverages were served from a sealed container at a temperature between 10–12 °C over the 30 min drinking period.

### 2.4. Test Protocol

**Figure 1:**
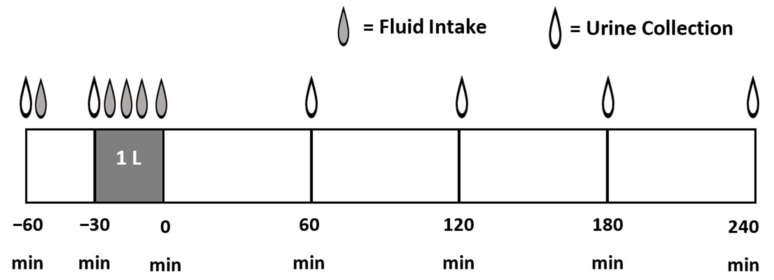
Schematic of the Beverage Hydration Index test protocol performed for each beverage trial (*n* = 4).

A schematic of the BHI test protocol is depicted in Figure 1, based on the protocol similar to that described by Maughan et al. [15]. Prior to the first experimental trial, a baseline determination of euhydration based on body mass and urine-specific gravity was performed. All experimental sessions were completed in a temperate (~21°C) room. Participants consumed a similar habitual food and fluid intake (as shown in Table 3) and avoided exercise within 24 h prior to each test session. To establish euhydration prior to the experimental sessions, participants were requested to consume an additional one liter of water between 1800 and 2300 h in addition to their normal daily intake and then fast (food and fluid) until morning. Tap water was provided (500 mL in opaque container) for participants to consume no more than 60 min prior to beginning the laboratory experiment (with the time of ingestion noted and repeated prior to each trial). Participants emptied their bladder upon waking, retaining a mid-stream sample in a sterile collection cup provided to them. Urine-specific gravity was measured by refractometry (Atago, URC-Ne Refractometer, Tokyo; Japan) and found to be similar across the four beverage trials (mean values <1.020).

Participants were given one liter of a test beverage during a test session while at rest: W, E, C + E, and AG + E over 30 min (four equal aliquots ingested every 7.5 min). After ingesting the beverage, participants remained seated, measured their nude body mass, and completed a beverage rating form. Beverage palatability, acceptability, and gastrointestinal comfort were assessed using a 10 m visual analog rating scale (VAS). The VAS measures attitudes across a continuum to provide continuous data based on two opposite verbal anchors (as compared to a categorical Likert rating scale). Subjects place a vertical mark at a point between the two verbal anchors to reflect their perceptions. The post-ingestion observation period was 240 min in duration after drinking the one-liter bolus. Subsequent measures (urine mass, body mass) were obtained at 0, 60, 120, 180, and 240 min post ingestion, similar to previous studies [15,19]. If participants needed to void between each hourly measurement (particularly during the first 120 min), the additional collection was counted toward the cumulative mass within that 60 min period. There was no food provided for the entire test session.

Nude body mass and urine mass was measured on a precision digital platform scale (Model DS100, Wiggins Scale Company, Atlanta, GA, USA). The men collected urine directly into a 3L plastic 24h urine container (Parter Medical Products, Carson, CA, USA), while the women utilized Vakly Graduated Specimen Collector Pans (Avaline Medical LLC, Lakewood, NJ, USA) placed under the toilet seat. The cumulative mass across each serial time point was measured on an electronic balance (to the nearest 0.1 g), with the mass of the empty container tared in order to subtract from total urine mass. The BHI was calculated as the ratio of cumulative urine mass for deionized water divided by the cumulative urine mass of the test beverage at each time point, as originally described [15]. In addition, participants provided at least 10 mL of urine in a specimen cup to allow for serial measures of urine osmolality (with this mass added to the total). Urine osmolality (UOsm) was measured in 50 µL samples in triplicate with the freezing point depression method (Micro-Osmette; Precision Systems Inc., Natick, MA, USA). The median of three values was reported, but up to five samples were run if values deviated by >3 mosmol/kg. All urine measures were performed on fresh samples at room temperature (not previously refrigerated or frozen). Prior to each session, the osmometer accuracy was verified using known reference solutions between 100 and 500 mosmol/kg (Osmolality Linearity Set, Advanced Instruments Inc., Norwood, MA, USA). Net fluid balance was calculated based on individual changes in body mass relative to the drink volume ingested.

### 2.5. Statistical Analysis

A two-factor analysis of variance with repeated measures (beverage × time) was used. Data were analyzed using SPSS version 26 (IBM SPSS Statistics for Windows, Version 26.0. Armonk, NY, USA: IBM Corp). Post-hoc multiple comparisons were corrected using the Bonferroni method. An alpha level of *p* < 0.05 was chosen to denote statistical significance. Data are presented as mean (±SD) unless indicated otherwise.

Our effect-size calculations utilized the data reported previously [15], indicating a minimum sample size for each test drink of *n* = 15. Although not a cluster randomized trial, we factored in an additional sample size weighting to account for possible increased variance due to combining men and women. The final sample size estimate based on 80% power with mean total urine output of 900 mL, pooled SD of 300 mL, and a mean difference of 220 mL, detectable at an alpha level of 0.05, required a total of *n* = 20 observations per drink. As stated previously, due to COVID-19 pandemic, we were only able to obtain complete data on 19 subjects. Previous studies [18,19] indicated no difference in BHI based on either sex or body mass; thus, both men and women were recruited. Although these previous studies found no significant sex-based effect, we could not establish equivalence with a 90% confidence level (likely due to the smaller number of women completing the study). Analyses were performed with and without the female subjects in the model. The model results did not change, and power was similar between analyses. Therefore, the fifteen men and four women were combined into a single group for statistical analysis.

## 3. Results

### 3.1. Beverage Hydration Index

**Figure 2:**
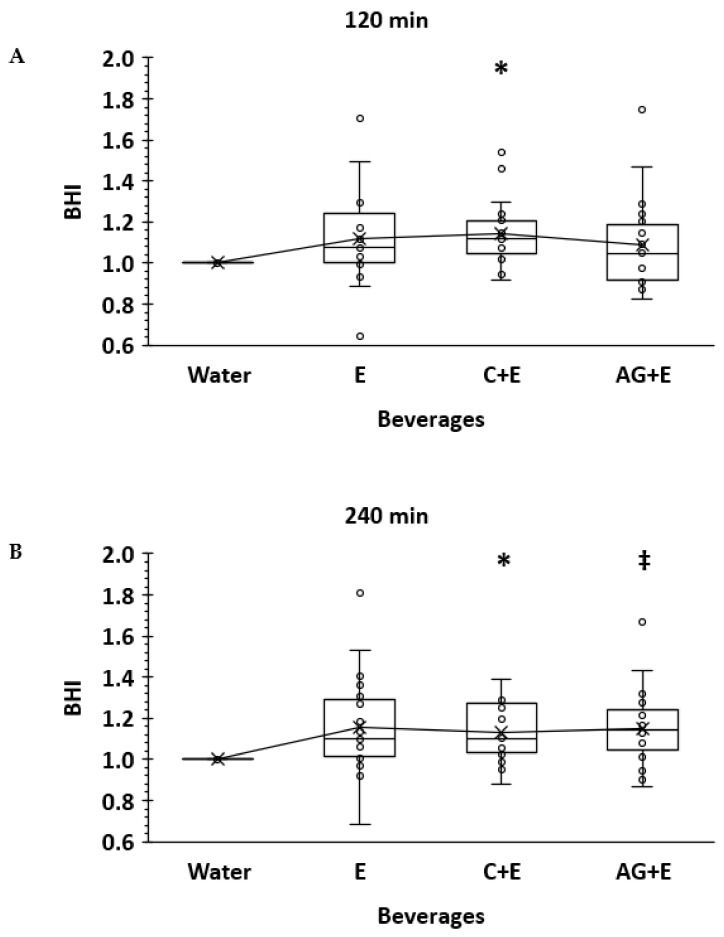
Box and whisker plot for beverage hydration index (BHI) at 120 min (**TOP panel A**) and 240 min (**BOTTOM panel B**) for test beverages and water (note: water is 1.0 standard). X = mean values connected by line across beverages; horizontal line = median value. * Carbohydrate-electrolyte (C + E) higher (*p* < 0.05) vs. Water; **‡** dipeptide-electrolyte (AG + E) higher (*p* < 0.05) vs. Water.

Figure 2 illustrates a box plot indicating the mean, median, and individual BHI values by beverage at 120 and 240 min. The main effect for beverage was not significant (*p* = 0.052), but there was a significant beverage × time interaction effect (*p* = 0.047). There was no significant change in BHI over time each hour following ingestion (*p* = 0.50), consistent with previous studies [15,19] in young adults, thus underlying the presentation of results highlighting 120 and 240 min. When compared to W, BHI for C + E was significantly higher (1.15 ± 0.17), with differences observed at 120, 180, and 240 min (*p* = 0.007, *p* = 0.018, and *p* = 0.006, respectively). AG + E was higher (1.14 ± 0.20) versus W only at 240 min (*p* = 0.021). Although on a trend to be higher compared to W, there were no significant differences for E at any time point, with BHI ranging from 1.12 ± 0.24 (*p* = 0.23) to 1.15 ± 0.25 (*p* = 0.09) at 120 and 240 min, respectively. None of the three test beverages (E, C + E, AG + E) differed from each other (*p* > 0.05) at any time over the 4 h post-ingestion period.

**Figure 3:**
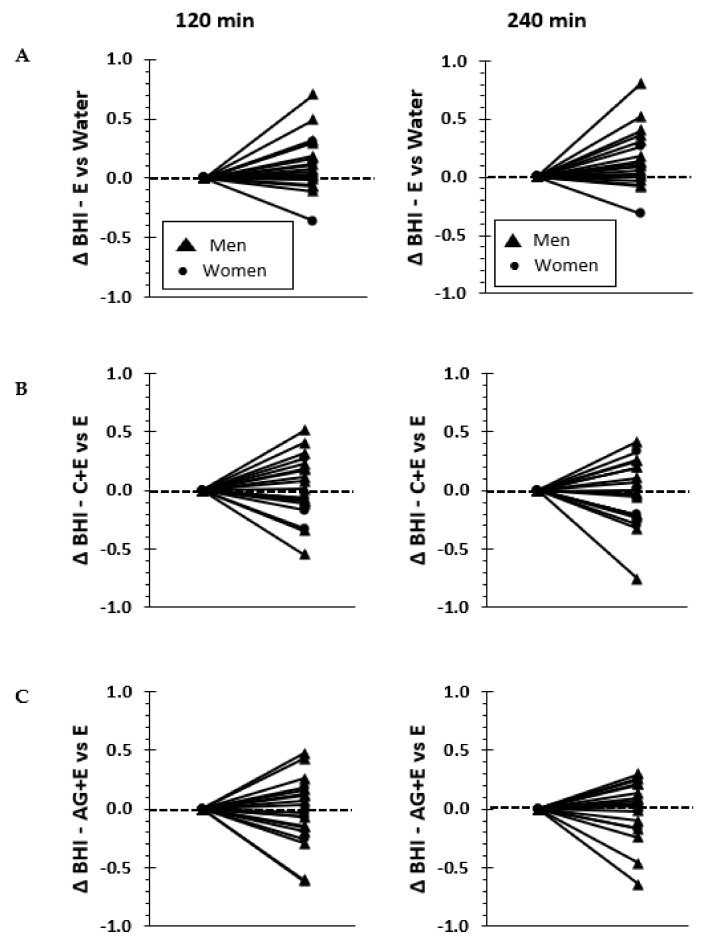
Individual subject data illustrating the net change in Beverage Hydration Index (BHI) relative to water (versus electrolytes) in **Panel A**; relative to electrolyte base solution (versus carbohydrate added) in **Panel B** and relative to electrolyte base solution (versus dipeptide added) in **Panel C** at 120 min (left columns) and 240 min (right columns).

Individual responses for the net change in BHI are presented in Figure 3. To understand the potential additive effects of electrolytes, carbohydrate, and protein to the BHI, pairwise individual net differences in BHI are shown using either W (to compare the net effects of E) or E (to compare the net effects of adding either C or AG) as the reference value. The mean (±SD) net differences in BHI for E versus W were 12 and 15% higher (0.120 ± 0.24 and 0.156 ± 0.25) at 120 and 240 min, respectively. However, the mean net effect was only 3% higher at 120 min (0.026 ± 0.27) when adding C to the E base solution and was significantly lower (*p* = 0.03) at 240 min (−0.026 ± 0.28). Furthermore, adding AG did not enhance the net difference in BHI (−0.03 ± 0.29 and −0.005 ± 0.25) relative to E either at 120 or 240 min. Pairwise comparisons of the overall mean net differences in BHI were not different among beverages (*p* < 0.16).

### 3.2. Net Fluid Balance, Urine Mass, and Osmolality

**Figure 4:**
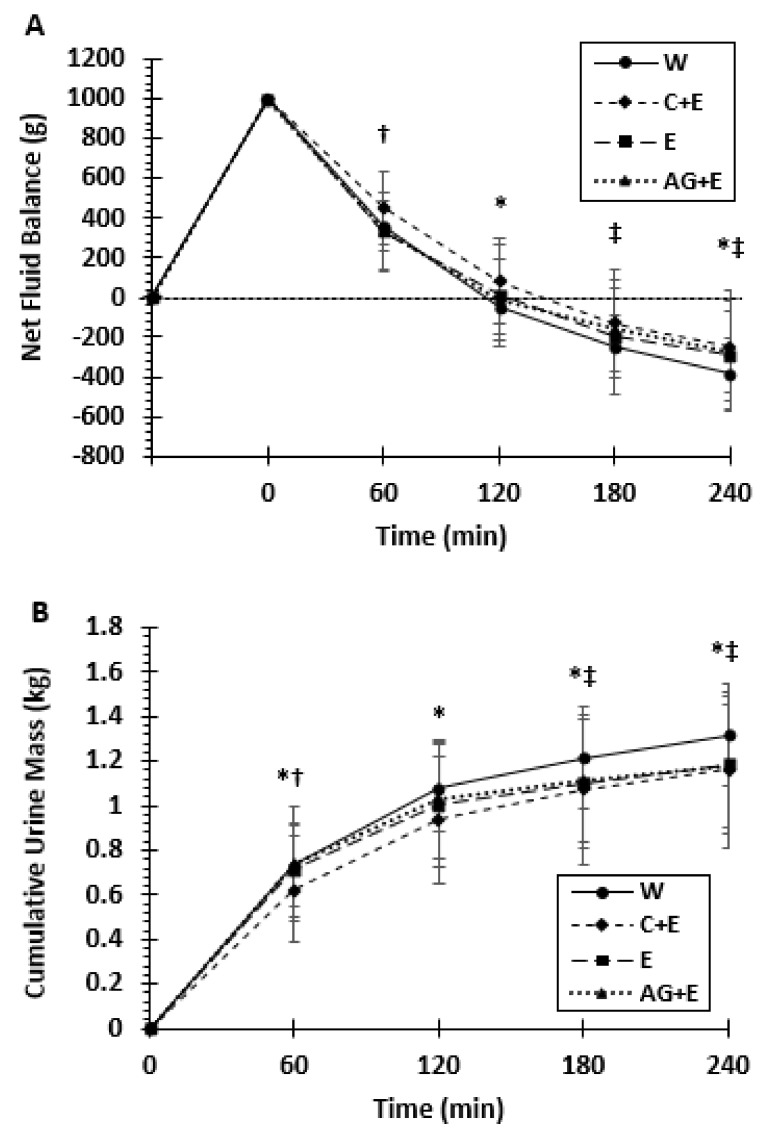
Mean (±SD) net fluid balance (**A**) and mean (±SD) cumulative urine mass (**B**) over 240 min based on beverages W (water); E (electrolyte); C + E (carbohydrate + electrolyte); and AG + E (dipeptide+ electrolyte). * Difference between W vs. C + E; † between AG + E vs. C + E; ‡ between W vs. AG + E (*p* < 0.05).

Figure 4A illustrates the change in net fluid balance over 240 min following ingestion of each beverage. There was no main effect for beverage (*p* = 0.17), although the mean fluid balance averaged over all time points tended to favor C + E by 120 mL versus W. There was a significant beverage x time interaction effect (*p* = 0.027). At 60 min, C + E yielded greater (*p* = 0.035) net fluid balance compared to AG + E. Compared to W, C + E elicited greater (*p* = 0.048) net fluid balance at 120 and 240 min and AG + E higher at 180–240 min. However, as depicted in Figure 4A, only C + E and E provided a positive net fluid balance (above 0) at 120 min post ingestion. 

Cumulative urine mass is plotted in Figure 4B over 240 min. The main effect for beverage was not significant (*p* = 0.072), but there was a significant beverage x time interaction (*p* = 0.004). The overall urine mass was lower (*p* = 0.02) for C + E vs. W at each time point. Urine mass was also lower (*p* = 0.03) for AG + E compared to W but only at 180–240 min.

**Figure 5:**
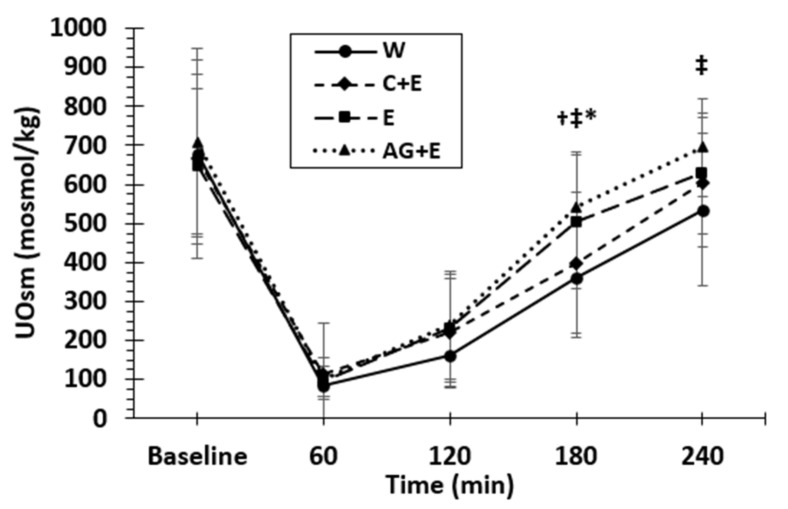
Mean (±SD) urine osmolality (UOsm) over time denoting significant differences between beverages. **‡** Difference between dipeptide-electrolyte (AG + E) vs. water (W); * Electrolyte (E) vs. W; **†** AG + E vs. carbohydrate-electrolyte (C + E) (*p* < 0.05).

UOsm is plotted over time in Figure 5. There were both significant main beverage (*p* = 0.005) and beverage by time interaction (*p* = 0.006) effects. Both AG + E and E elicited higher (*p* < 0.001) overall urine osmolality (by 112 and 82 mosmol/kg, respectively) versus an average UOsm for W (294 ± 25 mosmol/kg). There was no difference in urine osmolality among beverages upon the first morning void (baseline), but differences emerged after 180 min post ingestion in AG + E and E (*p* = 0.009) compared to W, and AG + E was also higher (*p* = 0.027) than C + E. By 240 min, only AG + E remained higher (*p* = 0.025) compared to W with no other differences among beverages.

### 3.3. Beverage Ratings, Tolerance, and Acceptability

**Figure 6:**
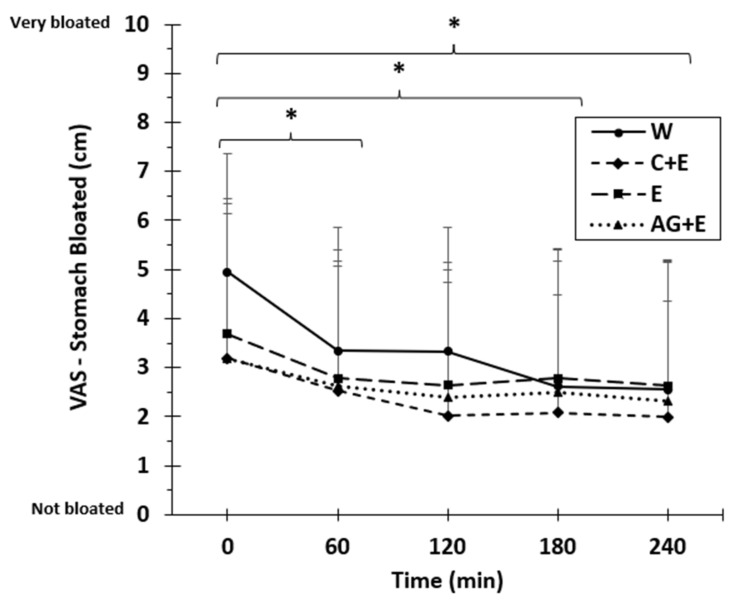
Mean (±SD) visual analog scale (VAS) ratings for stomach bloating after ingesting 1 L of beverages: W, water; E, electrolyte drink; C + E, carbohydrate-electrolyte sports drink; AG + E, dipeptide-electrolyte drink. * Difference (*p* < 0.02) for W at 60, 180, and 240 min compared to immediately after ingestion (0 min).

Table 4 illustrates the VAS subjective ratings of beverage palatability, tolerance, and acceptability, which were obtained immediately following the 30 min drinking period. W was rated lower than the three test beverages for sweetness (*p* = 0.005), saltiness (*p* = 0.01), and overall taste (*p* = 0.002). There was no difference (*p* > 0.05) in these ratings among E, C + E, or AG + E. There were no differences for gastro-intestinal tolerance or acceptability except in the rating for stomach bloating. Ratings for stomach bloating on W were higher immediately after the post-ingestion period (*p* = 0.02) compared to C + E and AG + E. Figure 6 indicates the rating for stomach bloating after drinking W was significantly lower (*p* = 0.018) after 60 and then again at 180 and 240 min, whereas the ratings for E, C + E, and AG + E did not change over time (*p* > 0.05).

## 4. Discussion

Our hypothesis that the addition of electrolytes to water improves fluid retention using the BHI model was only partially supported. Although the E solution (21 mmol Na^+^, 3 mmol K^+^) increased BHI by a higher value (>12–15% more fluid retention versus water), E was not statistically different from water in this group of young adults. This was likely due to individual variability in this trial, although E yielded a mean BHI consistent with the threshold value previously deemed to be of practical importance (>13%) in the literature [19]. The use of the BHI model to assess a beverage’s effect on longer-term hydration status and fluid balance is deemed of clinical and practical importance for the general population at large but in particular when either fluid availability or the ability to access bathroom facilities is limited [15]. When comparing the individual net differences in BHI from water, it is clear that the inclusion of electrolytes had the largest magnitude of effect on BHI.

The BHI for C + E also met this threshold [19] deemed of practical importance (>13–15%) relative to water over 240 min. This BHI is of a magnitude lower than that reported for ORS (Diarolyte) or milk (both skim and full fat), which were ≥1.5 (or 50% greater than still water) compared with the sports drink containing lower carbohydrate (3.9%) but similar Na^+^ (21 mmol). Maughan and colleagues [13] also reported increasing the carbohydrate content above 5% (to 10 and 20%) improved fluid retention of beverages using the BHI protocol. In the present study, the addition of 6% carbohydrate and/or modest inclusion of amino acids increased the BHI to be significantly different from water; however, the time course for a higher BHI was not in parallel. Compared to water, the C + E sports drink elicited a higher BHI earlier (by 120 min) compared to a dipeptide-containing beverage (which was not observed until 240 min). This was also supported by the calculation of the net difference effect on BHI for C + E relative to E, being significantly higher at 120 min compared to 240 min.

Although several BHI studies have already been published, a key question remaining centers around the minimal sodium requirement to improve fluid retention of a beverage under everyday sedentary conditions. Few studies have compared an electrolyte-only beverage (without macronutrients) in the literature. The lone study [13] to investigate the impact of graded levels of sodium content on BHI found that 27 and 52 mmol Na^+^ improved net fluid balance and accumulated less urine compared to 7 and 15 mmol Na^+^ solutions. However, our results did not find that a zero-calorie, electrolyte-only beverage (~21 mmol/L Na^+^) consistently improved BHI or the net fluid balance over water. Commercially-available beverages often considered to be ORS (e.g., Pedialyte) yield BHI values averaging > 1.2–1.5 [15,17,19], but this beverage category also contains macronutrients (e.g., ~2% carbohydrate) along with a higher range of sodium (dosing between 30–55 mmol). The WHO ORS formula recommends an even higher range with 75 mmol of Na^+^ [20]. A popular product that has become commercially available are additive electrolyte powders or tablets. Pence et al. [18] investigated the BHI using electrolyte tablets at two different concentrations. In this study, tablets were added to cold bottled water, each containing 2 g carbohydrate, 300 mg Na^+^, 150 mg K^+^, as well as calcium (13 mg), magnesium (25 mg), and chloride (40 mg). We calculated dosages (three or six tablets in 1.4 L water) of the two solutions to be approximately 28 and 56 mmol Na^+^ with carbohydrate <1%. Of note, the low dose was somewhat higher than the product package recommendation (two tablets per liter), equating to a ~26 mmol Na^+^, 0.4% carbohydrate solution. Interestingly, these authors report an insignificant overall beverage effect (*p* = 0.13) but go on to indicate only a significant contrast was found for the lower dose Na^+^ beverage versus water (and not for the double dose). The magnitude for the BHI values we report for E (above 1.1 but <1.2) were similar to this [18] and another study [17]. However, the finding that beverage sodium concentration was not predictive of BHI [18] is in contrast to others [13,17], who observed progressive increases in BHI directly related to beverage sodium content. Thus, it appears from the aforementioned studies that the minimal Na^+^ dose to elicit improved fluid retention is likely more than 21 mmol.

Another question related to BHI is the effect of the beverage energy density (e.g., carbohydrate). It follows that a lower absolute water content of a beverage would reduce the available fluid to effect volume expansion and subsequently diuresis compared to 100% water (thereby indirectly influencing markers of fluid retention). Maughan et al. [15] corrected for beverage water content and confirmed this alone did not seem to affect the BHI for a sports drink relative to water. In a more recent study [13], a higher carbohydrate content (10 and 20% vs. 0 and 5% carbohydrate) was needed to significantly increase BHI and was likely attributed to a slower fluid delivery. It has been documented that as the energy density of beverages increases, the appearance of water in the blood stream is reduced [27] as an overall byproduct of both gastric emptying and intestinal absorption. Thus, it stands to reason that less total fluid (94% wt/volume) in a sports drink combined with slower delivery into the blood might reduce diuresis particularly in the early post-ingestion period, consistent with our observations. 

The taste and sweetness ratings of the beverages indicate that the alanyl-glutamine dipeptide was similar to the sugar-based beverage and above that of plain water in the current study. This might confer advantages both in clinical populations (e.g., diabetic, obese) requiring adequate hydration but needing to restrict both calorie content and sugar as well as active healthy populations who need to replace daily sweat losses but not requiring the additional energy sources as compared to highly-trained athletes [28]. However, unexpectedly, we found greater ratings for stomach bloating immediately after drinking the 1 L of water compared to later in time (and higher compared to C + E and AG + E). Thus, the higher BHI we observed at 2 h for C + E may not necessarily be related to impaired gastric emptying but remains to be verified. Bloating, beverage taste, and acceptability are practical factors that influence ad libitum drinking patterns to maintain hydration. Whether adding electrolytes and/or certain macronutrients attenuate bloating when ingesting large volumes to rapidly replace fluid loss should be investigated further. 

Whether the level of carbohydrate commonly found in sports drinks (4–8% carbohydrate) improves, BHI appears to be equivocal. The BHI for a sports drink (with 3.9% carbohydrate, 21 mmol Na^+^) was originally reported as not different from water [15]. The sports drink in that investigation had 33% lower carbohydrate than in the current study (but similar electrolytes); however, a 6% sports drink was later confirmed [16,19] to be no different from water (BHI < 1.10) in contrast to other observations [17] and those reported here indicating a higher BHI for sports drinks in young adults. Moreover, adding carbohydrate (at 3, 6, and 12%) retained more fluid compared to placebo water without electrolytes over a 4-h post-exercise rehydration period, but the carbohydrate content per se did not impact fluid retention [7]. The reason for inconsistent conclusions regarding the BHI for a moderate carbohydrate sports drink relative to water are unclear but may be dependent upon individual differences in fluid availability and the delivery of beverages into the blood circulation while at rest. Peronnet et al. [29] validated that the time course for 300 mL of ingested water to reach peak blood levels (using an isotopic tracer of D_2_O) is highly variable among individuals. Thus, considering all these factors, it is likely that the minimum carbohydrate content to increase BHI may be ≥6% carbohydrate.

The type of carbohydrate found in a beverage may also be a factor influencing the BHI. Berry et al. [16] reported a milk permeate solution (devoid of fat and protein) with 4% glucose/galactose had a higher BHI (>1.2) compared to water, unlike a 6% sucrose/glucose sports drink. However, the fact that the milk permeate beverage had much higher mineral content (e.g., 28 mmol/L K^+^) and nearly 300 mosmol/kg higher osmolality (with contributions from magnesium, phosphorous, calcium, and chloride) despite a similar sodium and lower carbohydrate makes it difficult to clearly differentiate the impact of the carbohydrate type on BHI. Moreover, the influence of additional beverage minerals (e.g., potassium) on BHI remains obscure since increases in blood potassium influence aldosterone secretion and other physiological responses to maintain K^+^ balance, including both K^+^ excretion along with muscle cell K^+^ re-uptake [30].

Finally, the unique contribution that types of protein play in beverage hydration properties in normal daily life remains uncertain. Milk (skim and full fat) had a significantly higher BHI [15], much like ORS. Evidence from rehydration studies following exercise also suggest benefits in fluid retention, concluding that at a similar gram weight/volume, adding milk protein was more effective than carbohydrate [31]. At least three studies [14,17,19] have evaluated carbohydrate-free solutions with amino acids that report BHI above that of water (although not all were different from carbohydrate-electrolyte sports drinks). The combination of the different amino acids used (isoleucine, valine, serine, tyrosine, threonine, glycine, lysine, aspartic acid) and the dosage (5–7 g/L) varied. However, the amino acids in these beverages were also combined with relatively high levels of sodium, either 30 or 60 mmol [14,17] or 55 mmol [19], that independently raise the BHI. No study, to our knowledge, has directly compared the impact of adding dipeptide to a control electrolyte base solution. In the present study, the finding of a higher BHI for AG + E relative to water (and not for E) cannot be explained by a higher sodium content of the beverage. Adding a modest level of dipeptide (2 g/L) had a delayed benefit on BHI, emerging later than C + E, but elicited a more hyperosmotic urine versus both water and C + E sports drink by 180 min. Whether AG + E facilitated a faster absorption/fluid delivery than C + E cannot be ascertained in the present study despite the fact that, at 60 min, urine loss was greater (and net fluid balance lower) for AG + E versus C + E. Whether L-alanyl glutamine is unique in this regard is also unclear, although a recent review [32] cites strong evidence for amino acids to be protective of intestinal barrier function (acute and chronic supplementation in animal models), with clinical research emerging to support glutamine and arginine. According to Broer [33], negatively charged amino acids bring in 3 moles of sodium per 1 mole amino acid, suggesting that those amino acids would enhance fluid absorption to a greater extent than positively charged amino acids like arginine, which are not co-transported with sodium. Previously, L-glutamine or L-alanyl glutamine were suggested to have benefits in absorption for clinical models of diarrhea [21,22] and potentially following exercise [23], as it is transported across the brush border by a sodium-dependent mechanism. The alanyl-glutamine dipeptide is also more bioavailable than glutamine alone when matched for glutamine content, as evidenced by generating a greater increase in plasma glutamine [34]. This is likely due to a significant amount of free glutamine being readily oxidized by enterocytes [35]. However, the dosage of AG + E used in the present study was slightly lower compared to others [19] but similar to one (1 g/500 mL) that improved basketball shooting performance (unlike water) following dehydration [36]. Notwithstanding, based upon the available evidence, recommendations for the type or form of protein and amount to be included within an electrolyte formula to elevate the BHI appear premature and remain to be established.

### Limitations

Due to the global pandemic, we had an unbalanced study design with relatively few women and lower overall subject numbers than originally planned. Therefore, the generalizability of our results are likely limited to primarily young adult men. The BHI model by nature also does not allow for the evaluation of intestinal absorption rates of beverages to be interpreted within the context of urine output. Whether decreased urine output (and a higher BHI index) is influenced more by greater water retention or a slower entry of water across the gastrointestinal tract (particularly within the first hour following ingestion) cannot be ascertained. The clinical relevance for using the BHI model to apply directly to situations arising from severe dehydration (e.g., from excess sweat loss or prolonged fluid restriction) and/or clinical illness may also be questioned. Such conditions potentially result in endocrine perturbations and/or changes to the gut that may alter absorptive and renal responses to beverages. However, since these clinical conditions are difficult to control and replicate within the experimental setting, the use of the BHI model allows for a well-controlled assessment of pre-experimental hydration status of subjects in order to evaluate a longer-term fluid balance directly due to beverage composition (e.g., when speed of hydration may be less critical). 

## 5. Conclusions

In summary, the addition of electrolytes (in ranges typically found in sports drinks) may not significantly improve the BHI index per se but appear to make the greatest contribution in elevating the BHI compared to the addition of macronutrients. Furthermore, there was no meaningful difference among beverages in their short-term hydration status when electrolyte concentrations were equivalent. However, when compared to water, the timeline for improved fluid retention occurs earlier for a carbohydrate sports drink compared to a dipeptide beverage with similar electrolyte levels, and both beverages resulted in less acute stomach bloating when consumed in a large volume.

## Figures and Tables

**Table 1:** Mean (±SD) Subject Physical Characteristics.

	Men (*n* = 15)	Women (*n* = 4)	Total (*n* = 19)
Age (years)	25.3 ± 6.1	22.8 ± 2.1	24.7 ± 5.6
Height (cm)	180.0 ± 7.2	169.5 ± 5.7	177.8 ± 8.1
Weight (kg)	79.1 ± 9.5	67.2 ± 13.4	76.6 ± 11.2
% Body Fat—Skinfold	12.5 ± 4.9	24.9 ± 7.0	15.1 ± 7.4
Body Mass Index	24.4 ± 2.5	23.6 ± 5.3	24.3 ± 2.9
Fat Free Mass (kg)—Skinfold	69.3 ± 8.5	49.7 ± 6.0	65.2 ± 11.4
Total Body Water (kg)—Skinfold	52.3 ± 6.3	38.1 ± 6.1	49.9 ± 8.2
Total Body Water (kg)—InBody	48.7 ± 5.8	33.8 ± 2.1	46.2 ± 7.8

**Table 2:** Test beverage composition (measured values after conclusion of study).

Beverage	Osmolality (mOsm/kg)	Sodium (mmol)	Potassium (mmol)	Energy (kcal/L)
Water	2.2 ± 1.9	0.3 ± 0.1	0.5 ± 0.3	0
C + E	441.8 ± 0.8	21.3 ± 0.7	2.9 ± 0.1	232
E	87.6 ± 0.5	21.7 ± 0.0	3.1 ± 0.0	0
AG + E	94.8 ± 1.6	22.9 ± 0.4	3.3 ± 0.0	8

C + E = Carbohydrate-electrolyte, E = electrolyte, AG + E = dipeptide-electrolyte.

**Table 3:** Mean (±SD) 24 h dietary records prior to each beverage hydration index trial (*n* = 19). None of the nutrient intakes were different (*p* > 0.05) by treatment.

	WATER	C + E	E	AG + E
Water (g)	2487 ± 1290	2785 ± 1254	2616 ± 1452	2815 ± 1118
Energy (kcal)	2166 ± 691	1943 ± 713	1906 ± 536	2057 ± 582
Sodium (mg)	3375 ± 1423	3082 ± 1814	2948 ± 1448	2801 ± 1067
Protein (g)	116 ± 54	86 ± 43	109 ± 62	116 ± 61
Carbohydrate (g)	232 ± 80	231 ± 99	215 ± 80	222 ± 82

C + E = carbohydrate-electrolyte; E = electrolyte; AG + E = dipeptide-electrolyte beverages.

**Table 4:** Mean (±SD) beverage palatability, tolerance, and acceptability ratings on 10 cm visual analog scale.

	Water	C + E	E	AG + E
Bitter	0.9 ± 1.7	1.2 ± 1.4	1.6 ± 1.6	1.7 ± 2.0
Sweet	1.0 ± 1.7	6.6 ± 1.6 *	7.2 ± 1.4 *	6.8 ± 1.8 *
Sour	0.9 ± 1.7	2.2 ± 2.2	1.9 ± 2.1	2.0 ± 2.2
Salty	0.9 ± 1.7	2.1 ± 2.2 *	2.2 ± 2.3 *	2.2 ± 1.6 *
Taste	4.4 ± 1.4	7.1 ± 1.7 *	6.4 ± 1.3 *	6.8 ± 1.7 *
Thirst Quenching	6.5 ± 1.8	7.2 ± 1.1	7.0 ± 1.7	7.1 ± 1.5
Upset Stomach	2.9 ± 2.8	1.7 ± 2.3	2.3 ± 2.6	2.2 ± 2.7
Stomach Bloating	4.9 ± 2.4	3.2 ± 2.9 *	3.7 ± 2.8	3.2 ± 3.2 *
Nausea	1.5 ± 2.0	1.0 ± 1.8	0.8 ± 1.2	1.2 ± 1.8
Drink Again	7.1 ± 3.0	7.2 ± 2.6	6.0 ± 2.4	6.1 ± 2.9
Makes Me Feel Good	4.6 ± 1.5	5.9 ± 2.1	5.5 ± 1.2	5.8 ± 2.0

* Different from Water (*p* < 0.05). Carbohydrate-electrolyte (C+E), electrolyte (E) and dipeptide-electrolyte (AG+E) beverages.
